# On the Automatic Detection and Classification of Skin Cancer Using Deep Transfer Learning

**DOI:** 10.3390/s22134963

**Published:** 2022-06-30

**Authors:** Mohammad Fraiwan, Esraa Faouri

**Affiliations:** Department of Computer Engineering, Jordan University of Science and Technology, Irbid 22110, Jordan; enfaouri@just.edu.jo

**Keywords:** deep learning, skin lesions, skin cancer, melanoma, image classification

## Abstract

Skin cancer (melanoma and non-melanoma) is one of the most common cancer types and leads to hundreds of thousands of yearly deaths worldwide. It manifests itself through abnormal growth of skin cells. Early diagnosis drastically increases the chances of recovery. Moreover, it may render surgical, radiographic, or chemical therapies unnecessary or lessen their overall usage. Thus, healthcare costs can be reduced. The process of diagnosing skin cancer starts with dermoscopy, which inspects the general shape, size, and color characteristics of skin lesions, and suspected lesions undergo further sampling and lab tests for confirmation. Image-based diagnosis has undergone great advances recently due to the rise of deep learning artificial intelligence. The work in this paper examines the applicability of raw deep transfer learning in classifying images of skin lesions into seven possible categories. Using the HAM1000 dataset of dermoscopy images, a system that accepts these images as input without explicit feature extraction or preprocessing was developed using 13 deep transfer learning models. Extensive evaluation revealed the advantages and shortcomings of such a method. Although some cancer types were correctly classified with high accuracy, the imbalance of the dataset, the small number of images in some categories, and the large number of classes reduced the best overall accuracy to 82.9%.

## 1. Introduction

Skin cancer is considered one of the most dangerous types of cancer in the world [1,2], and the number of deaths is increasing daily as a result of this disease [3,4]. Moreover, it is one of the fastest spreading types of cancer [5]. However, treatment is possible if it is detected in its early stages [6]. According to recent statistics, it was reported that 20% of skin cancer reached a point where survival is not possible due to the disease progression [7]. Worldwide, approximately 50,000 people die each year from skin cancer [7,8], which represents 0.7 of the death rate due to cancer [8]. The estimated cost of treatment is approximately USD 30 million, which is prohibitive for treatment [5].

Doctors use multiple methods to detect skin cancer [9]. Visual detection is the initial way to identify the possibility of the disease [10,11]. The American Center for the Study of Dermatology developed a guide for the possible shape of melanoma, which is called ABCD (asymmetry, border, color, diameter) [2,12,13] and is used by doctors for initial screening of the disease. If a suspected skin lesion is found, the doctor takes a biopsy of the visible lesion on the skin [14], and examines it microscopically for a benign or malignant diagnosis and the type of skin cancer [15]. Dermoscopy is a technique that doctors use to diagnose skin cancer [16]. It involves taking bright pictures of the shape of the skin lesion, which comes in the form of dark spots [17]. However, this method faces many difficulties, the most important of which is the inability to determine the nature of the lesion due to the surrounding conditions such as the presence of hair, blood vessels, correct lighting, inability to take the correct shape of the spot, and the similarity of the shape of the spots among cancerous and non-cancerous diseases [18,19]. Moreover, some people may ignore skin lesions due to poverty, lack of access to proper healthcare, or misdiagnosis. Given an image of a skin lesion, the goal of this work to easily and automatically classify this image into benign or possible cancer. Such a system can be deployed as an easy-to-use smartphone application.

The contributions of this paper are as follows:

1. Develop an artificial intelligence-based screening system for skin cancer (melanoma and non-melanoma) using dermoscopic images of the skin lesions as input. Such a system can aid in clinical screening tests, reduce errors, and improve early diagnosis;
2. Implement transfer learning of 13 deep convolutional neural networks models for the classification of skin lesion images into seven categories, including melanoma, benign keratosis-like lesions, and five other non-melanoma cancers;
3. Evaluate classification performance using common relevant metrics for all models. In addition, the training behavior and time requirements were also included.

The remainder of this paper is organized as follows: the related work is discussed in Section 2, the dataset, deep learning models, and performance evaluation metrics and setup are explained in detail in Section 3, Section 4 presents the performance evaluation results along with a comparison to the related literature and discussion of the models, and we conclude in Section 5.

## 2. Related Work

Recent advances in artificial intelligence (AI) during the past decade and specifically in the field of deep learning and convolutional neural networks (CNNs) have opened the door for the development of reliable screening and diagnosis image-based medical systems [20]. The research landscape has recently witnessed a shift from image segmentation (i.e., separation of relevant areas in the image) and feature extraction toward automated classification using deep learning. The literature in the context of skin cancer detection/screening followed a similar trajectory with the traditional approach of image processing to remove irrelevant artifacts (e.g., hair) being overcome by using sophisticated deep learning artificial intelligence. Such recent techniques do not require explicit feature extraction and are generally immune to noise factors that affect images (e.g., light intensity, color, translation, reflection, etc.) [21]. However, they tend to be computationally intensive [22].

Li et al. [1] proposed digital hair removal (DHS) to filter the hair out of the skin lesion image, and analyzed the effect of hair removal using intra-structural similarity (Intra-SSIM). In another study, Liu et al. [23] developed a new method using deep learning to segment lesion images according to regions of interest (ROI). They used a new mid-level feature representation, where pre-trained neural networks (e.g., ResNet and DenseNet) were used to extract information from the ROI. Similarly, Pour and Seker [24] used convolutional neural networks for the segmentation of lesions and dermoscopic features. They used the CIELAB color space in addition to RGB color channels instead of excessive augmentation or using a pertained model. Almansi et al. [25] proposed a new segmentation methodology using full-resolution convolutional networks (FrCN). They worked on the image without pre/post-processing, and their results showed that the proposed method (FrCN) yielded better results than the other deep learning segmentation approaches. Dash et al. [26] proposed a new segmentation method based on a deep fully convolutional network comprised of 29 layers. Xie et al. [27] proposed the segmentation of dermoscopy images based on a convolutional neural network with an attention mechanism, which can preserve edge details. Serte and Demirel [28] proposed a novel Gabor wavelet-based deep learning model for the classification of melanoma and seborrheic keratosis. This model builds on an ensemble of seven Gabor wavelet-based CNN models. Furthermore, their model fuses the Gabor wavelet-based model and an image-based CNN model. The performance evaluation results showed that an ensemble of the image and Gabor wavelet-based models outperformed the individual separate image and Gabor wavelet-based models. This ensemble also outperformed the group of only Gabor wavelet-based CNN models.

Deep transfer learning has been widely deployed in the medical imaging literature for powerful, automatic, and internal (i.e., implicit) feature extraction. In this regard, Manzo et al. [29] employed a three-step approach for melanoma detection. In the first step, the images are converted into the proper size and the dataset is balanced. After that, deep transfer learning is used for feature extraction. These features feed an ensemble of traditional classification algorithms, including support-vector machine (SVM), logistic label propagation (LLP), and k-nearest neighbors (KNN). Jain et al. [30] compared six different transfer learning networks for multiclass lesion classification. However, their reported results relied upon increasing the size of the dataset by augmentation. Augmentation is typically used to introduce changes into the input images without duplication. Thus, making several augmented copies of the same image in the dataset will result in biased results that do not represent the actual performance [21].

## 3. Materials and Methods

**Figure 1:**
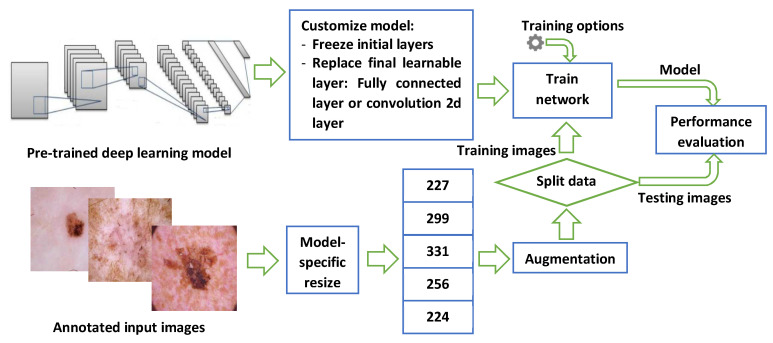
A graphical abstract of the general steps used in this paper.

Figure 1 shows the steps used to develop the skin cancer classification system using images of skin lesions. The methods used in this work do not need any feature extraction, nor does it perform any segmentation (i.e., separation of lesions from the rest of the image). All of these are automatically handled by the complexities of the deep learning model layers and operations. The next few subsections explain each part in detail.

### 3.1. Dataset

This work uses the dataset called HAM1000 (Human Against Machine) [2], which is comprised of 10,015 dermatoscopic images of the most common skin cancers. The images are divided into seven categories: 327 actinic keratosis and intraepithelial carcinoma (AKIEC), 514 basal cell carcinoma (BCC), 1099 benign keratosis-like lesions (BLS), 115 dermatofibroma (DF), 1113 melanoma (Mel), 6705 melanocytic nevi (NV), and 142 vascular lesions (VASC). Two augmentation operations were applied: random x-y scaling in the range (0.9, 1.1), and random x-y translation in the pixel range (−30, 30).

### 3.2. Deep Learning Models

Transfer learning has been found to be extremely effective in many image-based medical applications [31]. It replaces ad hoc deep convolutional neural network (CNN) designs with pre-trained, well-designed, and extensively-tested models. The initial layers of such models are trained to detect generic image features such as color, contrast, etc. On the other hand, later layers toward the output need to be customized and retrained on specific task-related features. Such methodology has proved its worth in a wide range of studies [20,22,32]. In this paper, 13 deep learning models were customized, retrained, evaluated individually, and compared on their ability to classify skin lesions into the seven aforementioned categories in the HAM1000 dataset. These were: SqueezeNet [33], GoogLeNet [34], Inceptionv3 [35], DenseNet-201 [36], MobileNetv2, ResNet18, RestNet50, ResNet101, Xception [37], Inception-ResNet, ShuffleNet [38], DarkNet-53 [39], and EfficientNet-b0 [40]. These models require input images to be of a certain size. More specifically, these models require the input to be of size 224 × 224 × 3, 227 × 227 × 3, 256 × 256 × 3, 299 × 299 × 3, or 331 × 331× 3. However, all of them were pre-trained using ImageNet [41].

### 3.3. Performance Evaluation Metrics and Setup

The performance was evaluated using five metrics [42]: accuracy, precision, recall, specificity, and F1 score. The accuracy measures the ratio of true positive plus true negatives for all the images. Precision measures the ratio of true positives to all elements identified as positives (including false positives). Recall (i.e., sensitivity) measures the ratio of true positives to all relevant elements (i.e., the actual positives). Specificity (i.e., selectivity) measures the ratio of true negatives to all images that are actually negative, and the F1 score is the harmonic mean of the recall and precision and expresses the accuracy of classification in unbalanced datasets. The five measures are defined in Equations (1)–(5). The reported results refer to the mean overall value when each separate class is considered as the positive case.

The model parameters were commonly set for all models as follows: minimum batch size = 16 (higher values are more computationally efficient but require significantly more memory), maximum number of epochs = 10 (no need to do further training if the loss/validation curve flattens out after a certain number of epochs with no improvement), initial learning rate = 0.0003, and the network solver = stochastic gradient descent with momentum (SGDM). Three strategies for data splitting into training and validation were used (i.e., 70/30, 80/20, and 90/10), which will measure the models’ improvement if more input images were available and their ability to generalize without overfitting the input images. Input images were augmented to increase their variety by using standard image processing operations as follows: random axis translation (i.e., image movement over the x and y axes) = (−30, 30), and random scaling using the range (0.9, 1.1).

The implementation and evaluation of the models was conducted using MATLAB R2021a software running on an HP OMEN 30L desktop GT13 with 64 GB RAM, an NVIDIA GeForce RTX 3080 GPU, an Intel Core i7-10700K CPU @ 3.80 GHz, and a 1TB SSD.

(1) $$Accuracy=\frac{TP+TN}{P+N}$$

(2) $$Precision=\frac{TP}{TP+FP}$$

(3) $$Recall=\frac{TP}{TP+FN}$$

(4) $$Specificity=\frac{TN}{TN+FP}$$

(5) $$F1=2\times \frac{Recall\times Precision}{Recall+Precision}$$

where TP represents the number of correctly classified images, FP represents the number of wrongly classified images as another class, FN indicates the number of images missed by the classifier, *P* indicates the number of all images considered as the positive class, and *N* is the number of all images other than the positive class.

## 4. Results and Discussion

The related work in the literature has already established that high performance is achievable in binary (i.e., benign vs. melanoma) or ternary (i.e., benign vs. melanoma vs. non-melanoma) classification of skin lesion images. The goal of the experiments was to evaluate the ability of transfer learning of the deep convolutional network models to correctly classify skin lesion images into one of the seven aforementioned categories in the dataset. Moreover, the training was repeated for 10 times to account for variability in the random data split of images into training and validation, and the mean values were reported. In addition, due to the high computational cost of deep learning models, the training and validation times were also included in the results.

**Figure 2:**
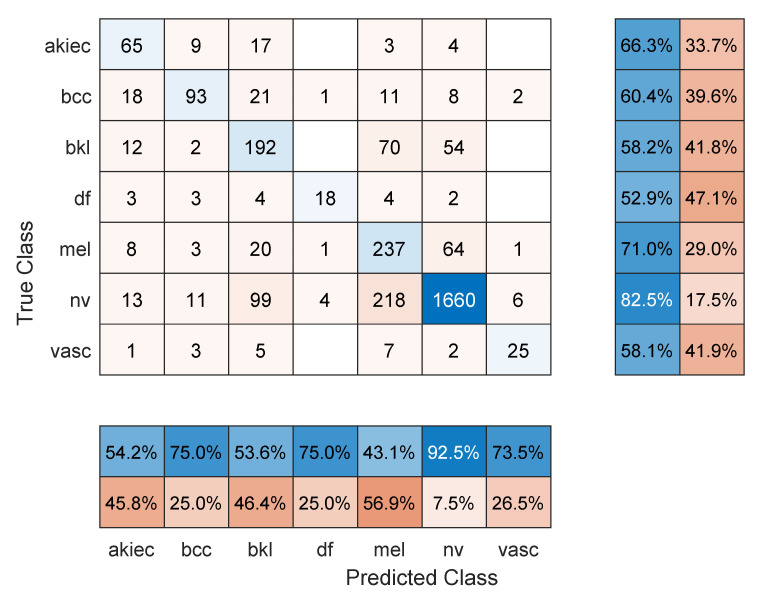
Sample confusion matrix for Resnet101 model and 70/30 data split.

Table 1 shows the mean overall performance metrics over 10 runs of each of the 13 deep learning models and using 70% of the data for training. All models achieved comparable accuracy values, with Resnet101 performing the best with 76.7%. The sample confusion matrix with row and column summaries in Figure 2 provides further insight into the results. First, due to the imbalanced number of images in each class and with smaller-sized classes achieving lower accuracies, the F1 score numbers are lower than the accuracy values. The NV class with the largest number of images achieved the highest precision (92.5%; see the NV column summary) and highest recall (82.5%; see the NV row summary). In comparison, the melanoma class was detected with 71% sensitivity (i.e., recall) but 43.1% precision. However, the other classes show less precision/recall variation.

**Figure 3:**
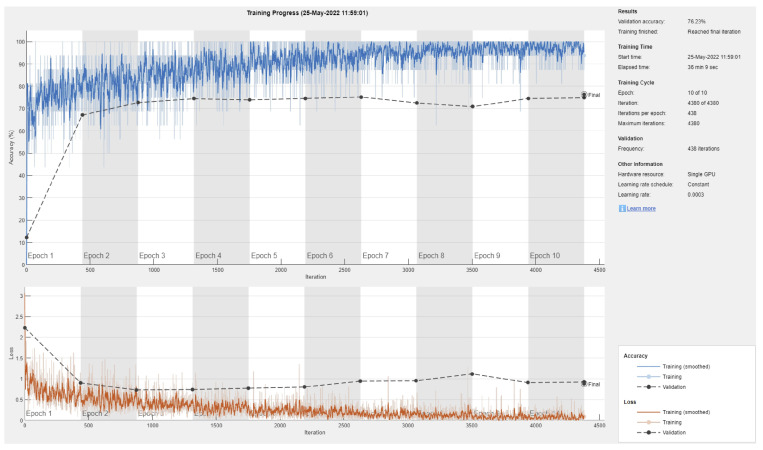
Sample training/validation progress curve for Resnet101 and 70/30 data split.

Figure 3 shows a sample training/validation progress curve for Resnet101 and a 70/30 data split. This figure shows two possible observations: first, the model is unable to achieve consistently reduced loss and produce high testing accuracy, even when the number of epochs is increased (not reported here), and second, due to the small number of images in most classes (deep learning requires large datasets [43]), there is an obvious gap between the validation vs testing performance (i.e., overfitting or inability to generalize to the validation data).

**Figure 4:**
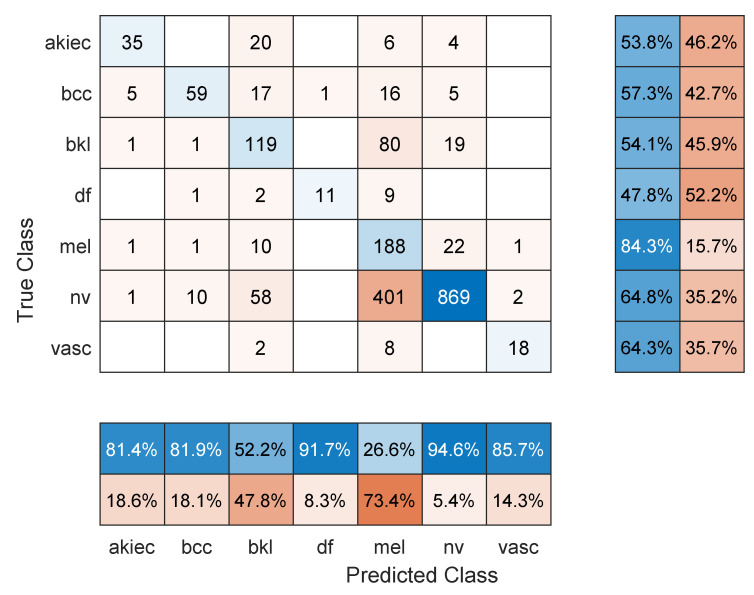
Sample confusion matrix for DenseNet201 model and 80/20 data split.

**Figure 5:**
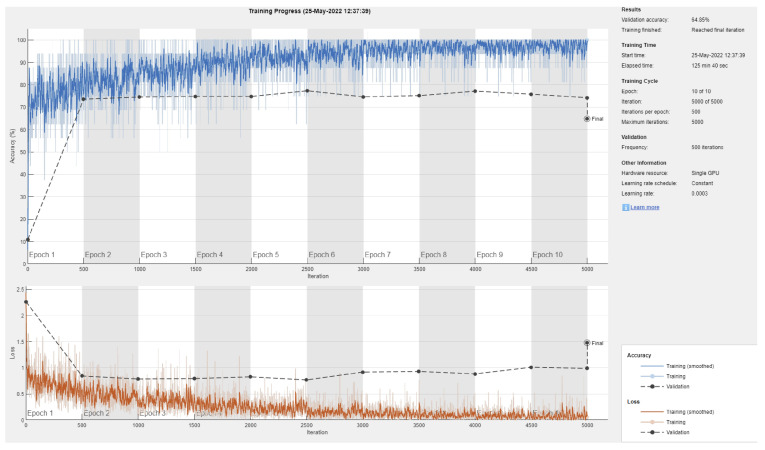
Sample training/validation progress curve for DenseNet201 and 80/20 data split.

Table 2 shows the mean overall performance metrics over 10 runs of each of the 13 deep learning models using 80% of the data for training. The 10% increase in the size of the training set did not have a significant effect on the performance metrics, with the best F1 score being 66.1% (DenseNet201 model). The confusion matrix in Figure 4 shows that a major source for errors was the misclassification of NV images as melanoma. Most classes achieved relatively high precision but low recall. Moreover, the same training and overfitting trends appear in Figure 5.

**Figure 6:**
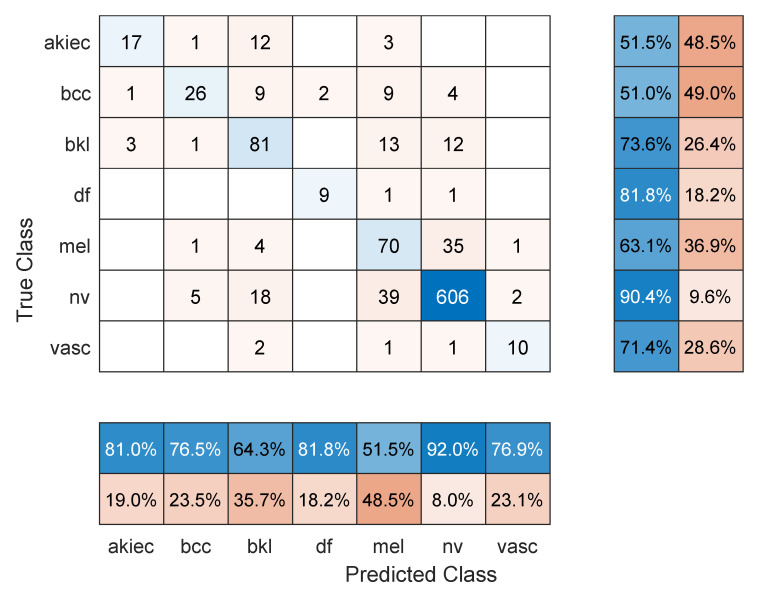
Sample confusion matrix for DarkNet-53 model and 90/10 data split.

**Figure 7:**
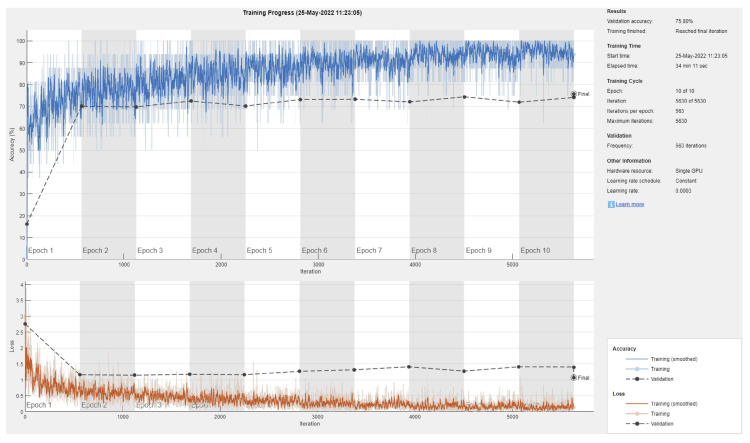
Sample training/validation progress curve for DarkNet-53 and 90/10 data split.

A further 10% increase in training data made the percentage of testing images 90% of the dataset. Table 3 shows the mean overall performance metrics over 10 runs of each of the 13 deep learning models. Three of the models (i.e., DenseNet201, DarkNet53, and ResNet101) achieved an accuracy above 80% with a corresponding F1 score of 74.4% for DenseNet201. The table shows steady improvement for most models with a larger set of training data over all metrics, except for the small model SqueezeNet. Generally, deep learning models, unlike traditional machine learning, benefit from larger datasets [44], which may be the reason for improved performance. The sample confusion matrix for DarkNet-53 in Figure 6 shows considerably better performance in terms of entries with one or fewer false misclassifications. However, the training/validation progress curve in Figure 7 still shows signs of overfitting.

Although an increased size of the training dataset showed signs of promise, much is still desired to reach a reliable diagnosis system that surpasses screening requirements. However, some of the results were affected by the small number of images in each class. For example, in Figure 6, the class DF had 11 images, VASC had 14 images, and AKIEC had 32 images. Such numbers are extremely low for an effective deep learning model, and single errors will have a profound effect on overall performance indices.

To assess the computational cost of training the deep learning models, the time required for each model was reported for each strategy of data split; see Table 4. In general, the required time increases linearly in less than 10% increments with each increase in the size of the training dataset. SqueezeNet is the fastest model, but DarkNet-53 is the best model that combines classification prowess with speed of training, followed by Resnet101.

A comparison to the related literature is shown in Table 5. Although the referenced studies achieve high performance values, they tackle a far easier problem in classifying fewer number of classes (two or three). Moreover, some of these studies require explicit feature extraction, which is not needed by deep transfer learning. Others, including Pezhman Pour and Seker [24] and Lie et al. [1], do not address the classification problem directly but rather on processing techniques for lesion segmentation (i.e., separation of lesion from other artifacts in the image) and hair removal from lesion images, respectively.

### Special Cases

**Figure 8:**
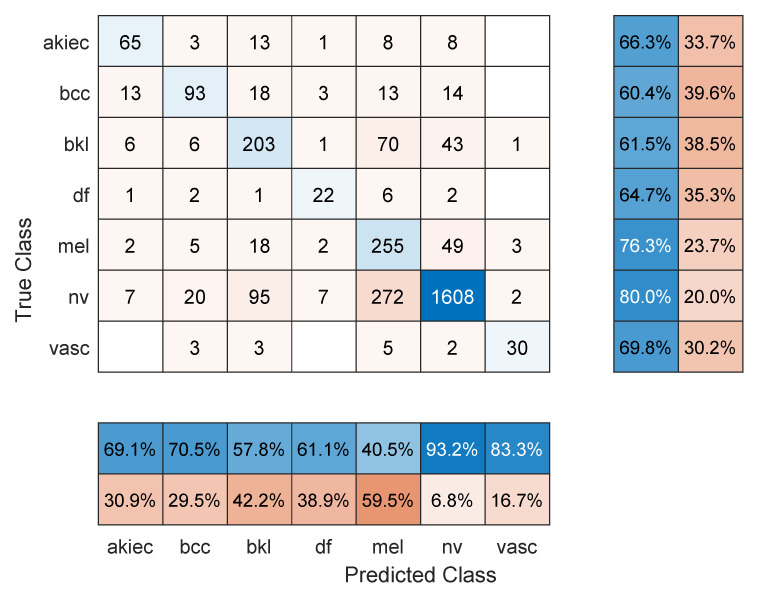
Sample confusion matrix for Resnet101 model, 70/30 data split and 50 epochs of training.

**Figure 9:**
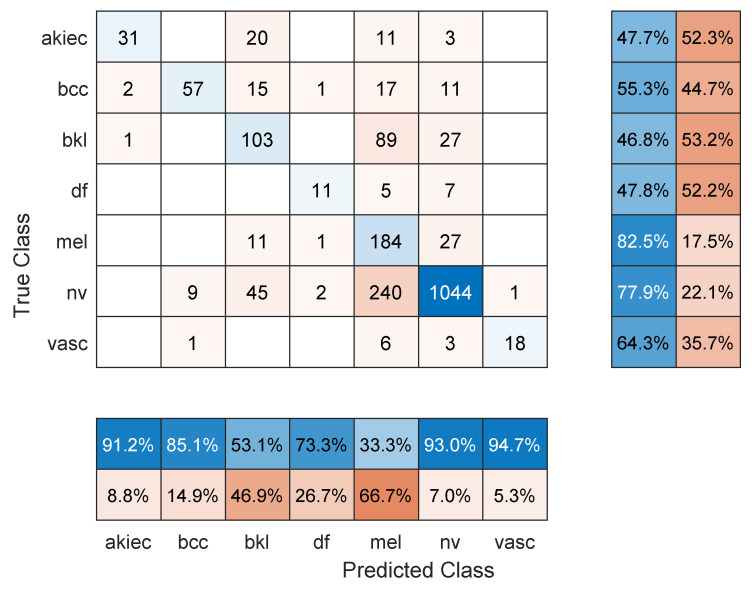
Sample confusion matrix for DenseNet201 model, 80/20 data split and 50 epochs of training.

**Figure 10:**
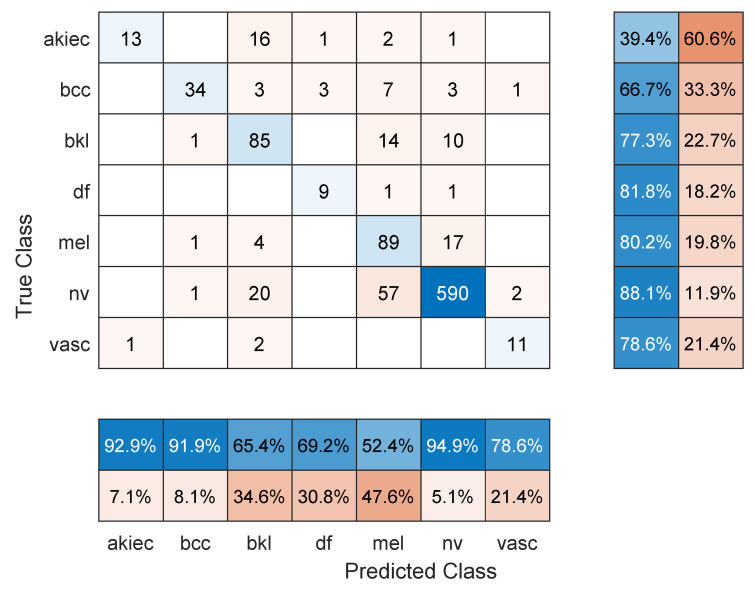
Sample confusion matrix for DarkNet-53 model, 90/10 data split and 50 epochs of training.

Further investigation of the classification performance and training behavior was conducted in order to shed light on shortcomings, as follows:

- Maximum number of epochs. Increasing the number of epochs will require more training time and may achieve better performance if the model has more room to learn, especially in large datasets. However, an exaggerated value for this hyper-parameter may lead to overfitting. Three models were retrained with a maximum number of epochs = 50. These were: Resnet101 with a 70/30 data split, DenseNet201 with an 80/20 data split, and DarkNet-53 with a 90/10 data split. In comparison to the values in Table 1, Table 2 and Table 3, the F1 score for Resnet101 improved slightly to 67.2% (was 64.3%), DenseNet201 performed a little worse with an F1-score of 63.7%, down from 66.1% in Table 2 (i.e., the model started to overfit the training data), and Darknet-53 improved to an F1-score of 83.1%. The other performance metrics showed similar trends to the F1 score. Figure 8, Figure 9 and Figure 10 show the corresponding confusion matrices;
- Classifying a lesser number of skin cancer types. Since the dataset is highly imbalanced with some classes having a significantly smaller number of images in the dataset (e.g., 115 DF and 142 VASC), it is worthwhile to explore several subsets of the classification problem as follows:
  − Eliminate the DF and VASC classes and perform 5-class classification. The same three models and corresponding data split as in the previous case with a maximum number of epochs = 10 were used. Surprisingly, in comparison to Table 1, Table 2 and Table 3, the F1 score displayed very small change (Resnet101: 64.8%, DenseNet201: 65.2%, and DarkNet-53: 67.1%), which was similar to the trend in the other performance metrics;
  − Eliminate the BCC (514 images), AKIEC (327 images), DF, and VASC classes and perform 3-class classification. The Resnet101 (70/30 data split), DenseNet201 (80/20 data split), and DarkNet-53 (90/10) were used with a maximum number of epochs =10. An easier classification problem has resulted in an improved F1 score for Resnet101 and DarkNet-53 of 71.1% and 72.8%, respectively. However, DenseNet201 performed worse at 62.3%, probably due to overfitting;
  − Using the same setup as above, perform pair-wise 2-class classification on the three classes, NV, MEL, and BKL. For the MEL vs. BKL classification, the F1 score of Resnet101 = 80.6%, DenseNet = 73.44%, and DarkNet201 = 83.7%. For the NV vs. MEL classification, all models performed badly. The F1 score for Resnet101 = 58.8%, DenseNet201 = 55.13%, and DarkNet-53 = 63.4%. Although the two classes have a good number of images, it seems like the similarities between the two types are too difficult to spot. Moreover, the lack of proper image cropping (i.e., elimination of useless parts of the images and keeping the lesion) contributed to this factor as it consumes a significant part of the image representation, especially that these algorithms require a scaled-down copy of the input, as mentioned in Section 3. The last pair-wise classification problem is NV vs. BKL, for which Resnet 101 achieved an F1 score = 72.8% (93% accuracy), DenseNet201 reported a 71.8% F1 score and 91.9% accuracy, and DarkNet-53 managed a 70.0% F1 score and 89.9% accuracy.

Surprisingly, lowering the number of classes did not result in improved performance in general. Although deep transfer learning has been effective in many medical and image-based applications, it seems like its application in this scenario requires more investigation and probably larger datasets.

## 5. Conclusions

Skin cancer in both melanoma and non-melanoma types is common and leads to many yearly deaths worldwide. Early diagnosis has been show to drastically reduce therapy time, cost, and suffering from the prolonged traditional treatment methods (e.g., chemotherapy). However, accurate screening/diagnosis requires specialist knowledge of the different types of cancers and how they appear in the form of skin lesions. Some people may ignore such lesions due to ignorance, indifference, cost, or doctor appointment scheduling delays. Recently, the field of deep learning and artificial intelligence has opened the door for the development of reliable image-based medical systems for screening and diagnosis. In this paper, we have used a well-known dermoscopy dataset of seven common types of cancerous skin lesions, utilized recent advances in the design of deep convolutional neural networks, and applied deep transfer learning to the application of screening/diagnosing skin lesion images. Such an approach has the capability to achieve high accuracies that reduce the burden on specialists. Moreover, it can be easily implemented and used in real-life applications due to the elimination of explicit feature extraction or manual image processing. Future work will focus on improving the balance of the dataset by collecting specific dermoscopy images of underrepresented skin lesion types and making those publicly available in the research domain.

## Figures and Tables

**Table 1:** The mean overall Accuracy, F-score, Precision, Recall, and Specificity for each deep learning model and 70/30 data split.

Model	F1 Score	Precision	Recall	Specificity	Accuracy
SqueezeNet	51.2%	63.1%	49.6%	93.8%	71.7%
GoogLeNet	55.4%	63.2%	53.4%	94.3%	74.0%
Inceptionv3	61.5%	65.5%	60.7%	94.5%	74.2%
DenseNet201	64.8%	70.9%	62.7%	94.7%	75.8%
MobileNetv2	61.0%	67.2%	58.4%	94.1%	75.6%
Resnet101	64.3%	67.6%	63.8%	95.0%	76.7%
Resnet50	63.4%	68.5%	62.4%	94.7%	74.4%
Resnet18	59.3%	64.7%	57.8%	94.6%	75.3%
Xception	60.9%	66.5%	59.2%	94.7%	75.4%
Inception-ResNet-v2	61.4%	65.3%	60.8%	94.4%	75.5%
ShuffleNet	60.6%	64.9%	58.7%	93.5%	74.6%
DarkNet-53	61.9%	66.8%	61.9%	94.5%	71.6%
EfficientNetb0	57.6%	70.3%	53.7%	94.1%	73.8%

**Table 2:** The mean overall accuracy, F-score, precision, recall, and specificity for each deep learning model and an 80/20 data split.

Model	F1 Score	Precision	Recall	Specificity	Accuracy
SqueezeNet	52.6%	64.0%	50.8%	93.4%	68.0%
GoogLeNet	56.2%	70.0%	53.8%	93.4%	68.5%
Inceptionv3	61.1%	64.2%	62.6%	94.0%	68.8%
DenseNet201	66.1%	74.7%	63.3%	94.3%	73.5%
MobileNetv2	61.5%	65.9%	60.2%	93.9%	73.0%
Resnet101	62.3%	69.0%	62.2%	94.2%	70.2%
Resnet50	63.2%	71.7%	61.8%	93.9%	67.7%
Resnet18	62.2%	64.7%	63.2%	93.8%	69.6%
Xception	56.1%	61.3%	55.9%	94.0%	70.2%
Inception-ResNet-v2	58.5%	63.9%	59.7%	93.8%	67.4%
ShuffleNet	61.2%	70.2%	57.8%	93.3%	70.0%
DarkNet-53	61.4%	70.7%	58.5%	93.6%	70.2%
EfficientNetb0	56.0%	69.8%	52.6%	93.6%	72.2%

**Table 3:** The mean overall accuracy, F-score, precision, recall, and specificity for each deep learning model and 90/10 data split.

Model	F1 Score	Precision	Recall	Specificity	Accuracy
SqueezeNet	52.7%	67.1%	48.0%	92.7%	75.0%
GoogLeNet	54.5%	64.2%	53.1%	94.5%	73.4%
Inceptionv3	67.9%	69.9%	70.1%	95.3%	79.3%
DenseNet201	74.4%	78.5%	73.6%	96.0%	82.9%
MobileNetv2	63.5%	68.8%	63.4%	94.8%	74.9%
Resnet101	71.7%	71.1%	74.5%	96.3%	81.2%
Resnet50	67.8%	72.6%	68.3%	95.5%	77.8%
Resnet18	67.9%	72.3%	68.3%	95.1%	79.0%
Xception	59.5%	65.0%	58.5%	94.4%	72.1%
Inception-ResNet-v2	64.4%	66.6%	66.8%	94.8%	73.9%
ShuffleNet	65.8%	74.0%	61.8%	94.3%	79.0%
DarkNet-53	66.3%	70.0%	66.1%	95.1%	80.8%
EfficientNetb0	61.3%	73.4%	57.0%	94.7%	76.7%

**Table 4:** The mean training and validation times for all algorithms and data split strategies. All times are in seconds.

Data Split	70/30	80/20	90/10
Model			
SqueezeNet	377.0	400.4	422.6
GoogLeNet	726.8	795	855.0
Inceptionv3	2182.9	2419.9	2655.2
DenseNet201	7190.8	7884.7	8686.6
MobileNetv2	3266.3	3678.5	4028.5
Resnet101	2196.5	2449.5	2682.7
Resnet50	992.2	1100.0	1192.9
Resnet18	413.6	439.9	470.0
Xception	9076.2	10,111.1	11,094.8
Inception-ResNet-v2	6698.0	7495.4	8254.3
ShuffleNet	2386.9	2641.0	2916.0
DarkNet-53	1761	1974.6	2126.3
EfficientNet-b0	5432.4	6028.4	6737.5

**Table 5:** A summary of the latest literature in automatic skin lesion classification.

Study	Objective	Dataset	Approach	Performance
Li et al. (2020) [23]	Two-class classification: melanoma and seborrheic keratosis	600 images	Mid-level features and segmentation according to ROI	Area under the receiver-operating characteristic curve, ResNet (89.00%), DenseNet (88.85%), Fusion(90.67%)
Pezhman Pour and Seker [24]	Lesion segmentation	3879 images	Dermoscopic feature segmentation using CNN	2% and 7% improvement in Jaccard index and sensitivity, respectively
Al-masni et al. [25]	Three-class classification: melanoma, benign, and seborrheic keratosis	2950 images	Segmentation using FrCN	Segmentation accuracy of 95.62% (clinical benign cases), 90.78% (melanoma, and 91.29% (seborrheic keratosis)
Dash et al. [26]	Three-class classification: moderate, severe, and very severe	6267 images	Segmentation using modified U-Net architecture	93.03% Dice coefficient, 94.8% accuracy, 89.6% sensitivity, and 97.60% specificity
Xie et al. [27]	Segmentation into two semantic classes: lesion and background	1479 images	Segmentation of dermoscopy images preserving edge details	Jaccard indices of 0.783, 0.858, and 0.857
Serte et al. [28]	Two-class classification: melanoma and seborrheic keratosis	2000 images	Gabor wavelet-based deep learning model for melanoma and seborrheic keratosis	Average area under the receiver-operating characteristic curve, 91%
Li et al. [1]	Optimal hair removal (reduce over/under removal)	1751 dermoscopic images with hair occlusion	Digital hair removal from images of skin lesion using CNN	Accuracy (99.08%), Specificity (99.85%), F1 score (94.43%), precision (99.09%), sensitivity (95.74%)
This work	Seven-class classification	10015 dermoscopic images	Deep transfer learning of a CNN	Accuracy (82.9%)

## Data Availability

Not applicable.

## Abbreviations

The following abbreviations are used in this manuscript:

ABCD	Asymmetry, border, color, diameter
AI	Artificial intelligence
CNN	Convolutional neural networks
DHS	Digital hair removal
Intra-SSIM	Intra-structural similarity
ROI	Regions of interest
CIELAB	International Commission on Illumination Lightness A, B
RGB	Red, green, blue
FrCN	Full-resolution convolutional networks
SVM	Support-vector machine
LLP	Logistic label propagation
KNN	K-nearest neighbors
HAM	Human against machine
AKIEC	Actinic keratoses and intraepithelial carcinoma
BCC	Basal cell carcinoma
BLS	Benign keratosis-like lesions
DF	Dermatofibroma
Mel	Melanoma
NV	Melanocytic nevi
VASC	Vascular lesions
TP	True positive
TN	True negative
FN	False negative
FP	False positive
N	Negatives
P	Positives
SGDM	Stochastic gradient descent with momentum
